# In-Vitro Assessment of Artificial Tooth Material Wear Behavior Using 3D Wear Analysis

**DOI:** 10.3390/jfb16070264

**Published:** 2025-07-16

**Authors:** Sıla Yelekçi, Ayben Şentürk, Funda Akaltan

**Affiliations:** 1 Align Technology, 06500 Ankara, Turkey; silayelekci@gmail.com; 2 Faculty of Dentistry, Prosthetic Dentistry, Ankara University, 06500 Ankara, Turkey; akaltan@ankara.edu.tr

**Keywords:** artificial teeth, nanohybrid composite, abrasion, chewing simulator, two body wear

## Abstract

Background: Artificial tooth wear impacts prosthesis durability and function; understanding material–antagonist interactions guides clinical choices. Aim: This in-vitro study aimed to assess the wear behavior of isosit and nanohybrid composite resin artificial teeth when opposed to various antagonist materials using 3D volumetric wear analysis. Materials and Methods: Sixty specimens (*n* = 10 per group) were prepared from two artificial tooth materials and assigned to six antagonist combinations: isosit–isosit, isosit–nanohybrid composite, isosit–porcelain, nanohybrid composite–isosit, nanohybrid composite–nanohybrid composite, and nanohybrid composite–porcelain. Specimens were scanned before and after 600,000 chewing cycles using a structured-light 3D scanner. Volumetric wear was calculated by superimposing pre- and post-test scans. Data were analyzed using two-way ANOVA and Tukey’s HSD test (α = 0.05). Results: Porcelain antagonists produced the highest wear values (*p* < 0.05). No significant difference was found between isosit and nanohybrid antagonists (*p* > 0.05). Identical material pairings showed less wear, though differences were not statistically significant. Conclusions: Porcelain as an antagonist increased wear risk. Using identical materials bilaterally, such as isosit–isosit or nanohybrid–nanohybrid, may help reduce artificial tooth wear in removable prostheses.

## 1. Introduction

In removable prostheses used for partially and completely edentulous patients, one of the most important properties of artificial teeth is their resistance to wear caused by opposing teeth and abrasive foods [1,2,3]. Wear resistance is defined as the ability of artificial teeth to remain in occlusion and maintain chewing efficiency for an extended period without undergoing dimensional changes [2,3,4,5].

Wear can be influenced by various factors, including abrasive foods, parafunctional habits, neuromuscular force, chewing patterns, antagonist material, and enamel thickness [6]. Furthermore, the microstructure, surface hardness, and durability of denture teeth are all influenced by the materials used in their manufacture, which makes them important factors in determining wear resistance [4]. Wear resistance may vary depending on the nature of the opposing material [7,8]. In cases of tooth loss, selecting an appropriate antagonist material for human enamel is essential. Ideally, to reduce tooth wear, artificial teeth should have similar properties to opposing teeth. One factor that increases the risk of tooth wear is the roughness of the outer surface of the antagonist material. Wear may also occur due to contact between tooth surfaces during dynamic occlusal movements [4,8,9,10].

The low wear resistance of artificial teeth is a major drawback of removable dentures since it prevents the denture from maintaining an appropriate occlusal relationship [1]. Additionally, artificial tooth wear can reduce efficiency in swallowing, speaking, and chewing [2]. Restorations are subjected to masticatory forces during functional and parafunctional occlusion, which can contribute to the wear of artificial teeth. Decreases in the occlusal vertical dimension may cause alterations in the functional performance of mastication and lead to muscle fatigue and ischemia [2,8,11].

In removable dentures, various materials, such as acrylic resin, composite, and ceramic have been preferred for the fabrication of artificial teeth [1,2,3,12]. Acrylic resin teeth have traditionally been preferred as artificial teeth in removable dentures due to their simple fabrication process and lower cost compared to porcelain teeth. One of their main advantages is their compatibility with the acrylic resin denture base. Furthermore, they are easy to reshape and polish [7,9,11,13,14]. However, acrylic resin teeth have relatively low wear resistance [6,15]. Porcelain teeth, on the other hand, are highly resistant to wear. Despite this advantage, they have significant drawbacks, such as difficulty bonding to the denture base, brittleness, and the potential to cause abrasion on enamel and other restorative materials due to their high hardness [3,5,14]. Other reasons for the preference for porcelain teeth are their color stability and aesthetic qualities [4,7].

To overcome the wear problem, considering the advantages of acrylic resin teeth, new types of acrylic resin teeth have been developed with different monomers, cross-linking agents, and inorganic fillers [2,6,13]. Interpenetrating polymer network (IPN) and double cross-linked (DCL) PMMA denture teeth are examples of denture teeth strengthened with a cross-linking agent [5,14]. Cross-linked acrylic resin teeth were developed to increase hardness and crack resistance; however, their bond strength to the denture base is weaker than that of conventional acrylic teeth [16]. Shortly after the introduction of cross-linked materials, urethane dimethacrylate (UDMA)-based teeth reinforced with inorganic micro-fillers were introduced [5]. Studies have shown that UDMA wears 40–50% less than IPNs [5,17,18]. Adding inorganic fillers to the polymer matrix can increase wear resistance [19], but some studies have reported different results, stating that no relationship exists between chemical structure and wear resistance [16]. Furthermore, similar to IPNs, UDMA exhibits greater porosity and color change than acrylic resin teeth and has adhesion problems with the denture base [20,21,22]. Composite resin teeth introduced in the 1980s became a better alternative to existing artificial teeth in terms of both wear resistance and aesthetics [23]. Studies have reported that vertical loss in composite resin teeth is lower than in both cross-linked and conventional acrylic resin teeth [6]. Notably, nano-hybrid composites introduced in 2009 have been reported to have excellent color stability and polishability [5]. However, studies on artificial teeth have not reached consistent conclusions [3,7]. Another important factor to consider when evaluating artificial teeth wear is the antagonist teeth. It has been shown that artificial teeth are undoubtedly influenced by the antagonist tooth material [23,24].

Quantitative wear measurements have been examined in numerous studies using non-contacting or contacting profilometers. Researchers recorded the vertical substance loss measurements of various profiles tested and averaged these values to obtain the mean two-dimensional step height. With the advancement of surface metrology software, 3D wear measurements have been used to record the wear loss (vertical and volume loss) of the wear scar. Both techniques are available in the laboratory and show a significant positive correlation. However, three-dimensional measurements are preferred over two-dimensional measurements as they allow for a more accurate evaluation of wear loss by providing a more precise analysis [6,13,25,26].

This study aimed to evaluate the wear of isosit and nano-filled composite resin artificial teeth against isosit, nano-filled composite, and porcelain materials using 3D wear measurement following a two-body wear test. The null hypotheses of this study were as follows: (i) different artificial teeth do not exhibit differences in wear when opposed to the same material; (ii) different antagonist materials do not affect the wear of the artificial teeth.

## 2. Materials and Methods

In this study, the 3D volumetric quantitative wear behavior of artificial tooth materials produced from two different materials (isosit and nanohybrid composite) were tested by using a dual-axis chewing simulator (Esetron, Smart Robotechnologies, Ankara, Turkey). The artificial tooth materials used in the study were identified into two groups as tested material and antagonist material. A total of 60 specimens were prepared, with 10 specimens in each group.

### 2.1. Tested Materials

1. Isosit (SR Orthosit PE, Lot 28578; Ivoclar Vivadent AG, Bolzano, Italy) and 2. Nanohybrid composite-NHC (SR Phonares II, Lot TP1444, Ivoclar Vivodent AG, Italy).

### 2.2. Antagonist Materials

1. Isosit (SR Orthosit PE, Lot 28578; Ivoclar Vivadent AG, Bolzano, Italy), 2. Nanohybrid composite-NHC (SR Phonares II, Lot TP1444, Ivoclar Vivodent AG, Bolzano, Italy), and 3. Porcelain (Synoform Lumin Vakuum, Lot M6; Vita Zahnfabrik, Bad Säckingen, Germany).

**Figure 1:**
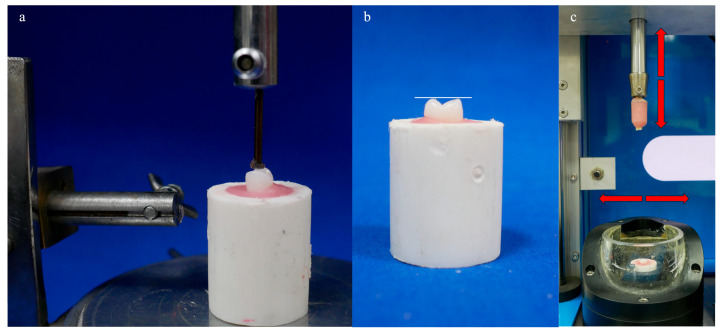
The artificial tooth specimens embedded in acrylic holders, (**a**) The specimen positioned in the acrylic block with the aid of a parallelometer, showing the alignment of buccal and palatal cusps at the same level. (**b**) The aligned view of the buccal and palatal cusps in the acrylic block. (**c**) The mounted artificial tooth specimens loaded in the chewing simulator for dynamic loading.

In the dual-axis chewing simulator, maxillary premolar artificial teeth were used in one jaw and mandibular premolar artificial teeth in the other jaw. These artificial teeth were anatomically similar in terms of size and shape. Using a paralellometer (Degussa AG, Hanau, Germany), the buccal and palatal cusps of the premolar teeth were aligned at the same level and embedded in auto-polymerizing acrylic resin (Meliodent; Kulzer GmbH, Hanau, Germany) holders within plastic molds (Figure 1a,b).

To standardize the tested artificial teeth surface, cusps of each tooth specimens were manually wet-abraded and finished with 2500 to 4000 grit abrasive paper (Silicone Carbide paper; Buehler GmbH, Düsseldorf, Germany) to a total depth of 0.5 mm with the aid of calipers, to obtain a flat area of approximately 2 mm × 3 mm for loading during the wear test.

After being placed in acrylic holders and having flat surfaces obtained on their buccal cusps, the dimensions of the artificial teeth were measured using a 3D laser scanning device (PC AC Omnicam, model no: 6390327; Sirona Dental Systems GmbH, Bensheim, Germany) before being placed in the chewing simulator.

In the wear test, the buccal cusps of mandibular first premolar artificial teeth were used as antagonists. The tested and antagonist specimens were mounted on a chewing simulator and subjected to 600,000 cycles (Figure 1c). The load weight for each antagonist was 5 kg, equivalent to an effective force of 49 N. This force value was selected, based on previous studies [6,27], to simulate high occlusal forces that may occur in situations such as bruxism or unbalanced occlusion. The specimens were tested in distilled water at 35.6 °C, similar to mouth temperature. The temperature of 35.6 °C was maintained throughout the test using a built-in temperature control system of the chewing simulator, which continuously monitored and regulated the temperature of the distilled water bath. In the simulated chewing motion, the vertical movement was set to 6 mm and the horizontal movement to 0.3 mm. The chewing simulators applied a load of 49 N with a frequency of 1.3 Hz over a total duration of 600,000 cycles, with vertical movement ranging from 0.2 mm to 6 mm. The selection of 600,000 cycles corresponds approximately to 3.5 years of clinical use in removable dentures, representing a period when functional occlusal wear becomes clinically relevant.

**Figure 2:**
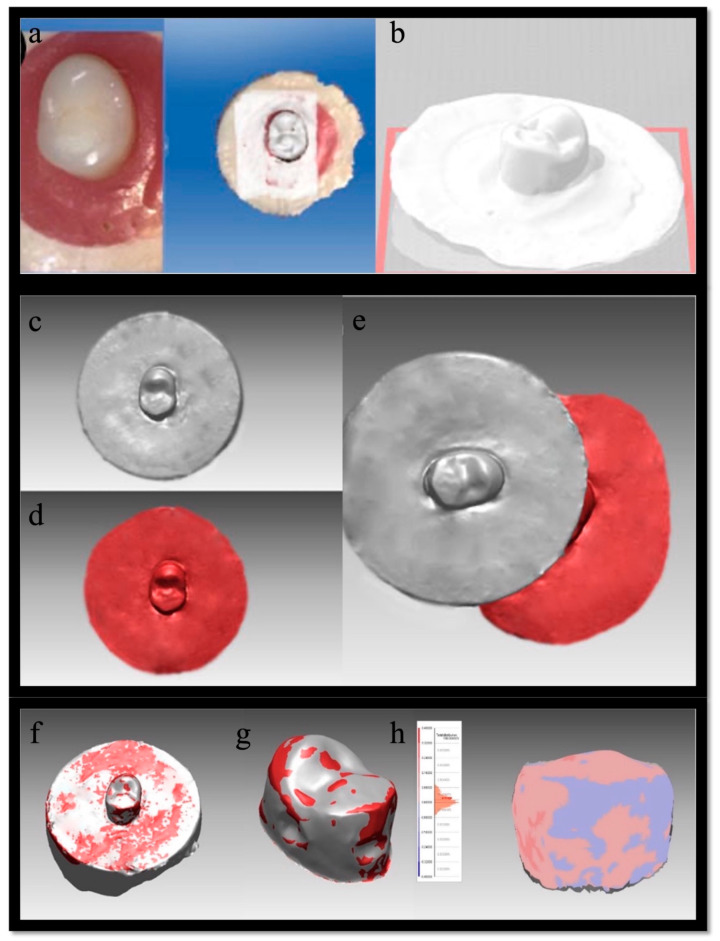
(**a**) Image of the artificial tooth specimen in the 3D laser scanning device, (**b**) STL image of the specimen, (**c**) the model obtained before wear was considered as fixed, (**d**) the other model obtained after wear, (**e**) superimposition of pre- and post-wear models using VrMesh software, (**f**) the superimposed artificial tooth specimen with the acrylic holder, (**g**) the specimen after being separated from the acrylic holder, (**h**) deviation map showing the differences between maximum and minimum volume values and color-coded 3D deviation highlighting wear areas.

After the completion of the chewing simulation, the images of the specimens were re-scanned using a 3D laser scanning device (PC AC Sirona Dental Systems, Cerec Omnicam, model no: 6390327, Bensheim, Germany) and recorded (Figure 2a). The STL (stereolithography) images (Figure 2b) obtained before and after the wear test were superimposed using VrMesh software (v11.2 VirtualGrid Inc., Bellevue City, WA, USA), and the volumetric difference between the two images was calculated (Figure 2c–e). The image of the artificial tooth specimens in the superimposed model was cut from the lower acrylic holder and prepared for the calculation of volume and deviation values (Figure 2f–h). The loading and measurement area on the buccal cusp and its surroundings were taken into consideration while measuring the deviation and volume in the artificial tooth specimens. Maximum, minimum, average, and standard distances on the surface were measured, and the volumetric values before and after the test were calculated and tabulated in an appropriate spreadsheet (Excel 2018 for Windows; Microsoft Corp, Redmond, WA, USA).

In the present study evaluating the volumetric wear behavior of artificial teeth, since there were two factors—tested and antagonist materials—the wear results were statistically analyzed using the Two-Way Factorial Analysis of Variance (ANOVA) with a significance level set at α = 0.05. Prior to performing the two-way ANOVA, the assumption of homogeneity of variances was tested using Levene’s test. The results confirmed that the variances were homogeneous (*p* > 0.05), validating the use of ANOVA for statistical analysis. Multiple comparisons of the significant differences between the mean wear values of the antagonist materials were assessed using the Tukey HSD multiple comparison test.

## 3. Results

A total of 60 specimens (10 per group) were prepared to assess the wear potentials of the isosit and nanohybrid composite (NHC) prosthetic teeth against isosit, NHC, and porcelain antagonists. During wear analysis, one specimen in each of the porcelain and isosit antagonist groups showed markedly deviating wear values (outliers), likely due to material heterogeneity or inconsistent antagonist contact. Outliers were identified using the [1.5 × IQR rule] and excluded from the final analysis to ensure data reliability. Statistical comparisons before and after exclusion indicated that removing outliers did not significantly alter the overall results or conclusions, confirming the robustness of the findings.

Wear values were calculated in mm^3^, and the descriptive statistics are presented in Table 1. Statistical analysis revealed a significant difference between at least two of the antagonist material groups (*p* < 0.05) (Table 2). Specifically, porcelain, as an antagonist material, resulted in significantly higher wear compared to other groups. However, no statistically significant differences were found between the tested artificial tooth materials (isosite and nanohybrid composite) or in the interaction between antagonist material and artificial tooth material (*p* > 0.05).

These findings indicate that the antagonist material has a more significant impact on wear than the type of artificial tooth material used. Porcelain antagonists led to the highest wear values (*p* < 0.05), while no significant differences were observed when Isosit or NHC was used as the antagonist material (*p* > 0.05). Although groups with similar combinations (isosite–isosite and NHC-NHC) tended to show lower wear than those with mixed combinations (isosite–NHC, NHC–isosite), the differences were not statistically significant (*p* > 0.05).

## 4. Discussion

This study aimed to evaluate the wear level of different types of artificial teeth using 3D wear measurement following a two-body wear test. According to the two-way factorial analysis of variance, only the antagonist material had a statistically significant effect on the wear level (*p* < 0.05), while the type of artificial tooth and its interaction with the antagonist material showed no significant effect (*p* > 0.05). Therefore, only the second null hypothesis—stating that different antagonist materials do not affect the wear of artificial teeth—was rejected.

Tooth wear is a complex and multifactorial process involving the interaction of mechanical, chemical, and biological factors within the oral environment. While in vitro studies like the present one provide controlled and standardized conditions for wear assessment, they cannot fully replicate the complex oral tribological system characterized by factors such as saliva composition, mastication dynamics, and individual behaviors [28]. Tooth wear can be evaluated through either in vivo or laboratory studies. Due to individual variability in factors, like salivary pH and oral temperature, laboratory studies offer more controlled and standardized conditions for wear testing [29,30,31]. In the present study, a chewing simulator was used to ensure consistent methodology [4].

DeLong and Douglas reported that 250,000 simulator cycles correspond to approximate one year of clinical use. Based on this, the 600,000 cycles employed in this study simulate approximately 3.5 years of wear in removable dentures, considering overnight removal [32]. Distilled water was used as a lubricant due to its consistency and effectiveness in facilitating movement and debris removal, as artificial saliva lacks standardization [33].

The applied load and cycle count significantly affect wear results. Suzuki et al. estimated that the occlusal force in monoplane dentures is around 23.2 N [34]. Some studies using lower loads (e.g., 5 N) found minimal wear [35]. A 49 N load was chosen in this study to simulate clinical conditions with high occlusal stress, such as bruxism or unbalanced occlusion [4,30].

The wear of artificial teeth can be studied in vitro using two-body or three-body testing methods. Three-body tests evaluate wear characteristics; however, results can be influenced by many variables, such as the abrasiveness and pH of the intermediate material, in addition to the antagonist material [23]. In contrast, direct contact between the test specimen and the antagonist material is demonstrated using the two-body wear testing method. This type of contact commonly occurs during swallowing and parafunctional habits [5]. Consequently, the two-body wear test method was selected for the present study.

In this study, wear measurement was conducted by performing 3D digital scanning of the specimens before and after wear, followed by the measurement of volumetric (3D) loss of the worn tooth structure through superimposition of the two 3D models. The vertical wear between the antagonist cusps and artificial teeth was not measured after the wear testing. In previous studies, vertical losses were measured in addition to volumetric loss, and similar results were obtained in both groups [6,7,23]. Other similar studies have demonstrated that 3D wear measurement is preferred over 2D wear measurements as it provides a more precise analysis of wear loss [4,27,36].

Hahnel et al. (2009) [23] demonstrated in their study that various wear mechanisms, ranging from minor surface grooves to severe material degradation, are influenced by the type of antagonist material. Therefore, it is not surprising that different antagonist materials lead to varying degrees of wear. These findings may explain the inconsistent data reported regarding the in vitro wear resistance of artificial resin teeth [7,16,35].

There are numerous studies that comparatively examine the wear of different artificial tooth materials [20,23,27,37,38]. In a wear test conducted on a fixed disk [39], no significant difference in wear resistance was observed between composite resin, porcelain, and conventional acrylic resin teeth, except for one group of acrylic resin teeth. However, the results of the present study are not consistent with previous studies that demonstrated higher wear resistance of composite resin teeth compared to conventional or highly cross-linked acrylic resin teeth [14,17,40]. On the other hand, the systematic review by Mudliar et al. [3] reported that acrylic teeth containing nano fillers do not show a significant increase in wear resistance, and therefore, nano-filled composites do not demonstrate superiority over conventional micro-filled or acrylic resin teeth. This finding is consistent with the results of our study.

In a study by Ghazal et al. (2008) [7], among the artificial teeth made of acrylic, nanofilled composites, and porcelain tested against both their own group and natural tooth antagonists, the highest wear values were observed in acrylic resin teeth. The same study found that porcelain artificial teeth exhibited the highest wear resistance against natural teeth. Nanofilled composite resin showed comparable wear values when tested against its own group, while acrylic resin teeth displayed higher wear than other combinations tested within their group. Although no statistical difference was found in total vertical substance loss between the nanofilled composite resin-natural enamel and feldspathic ceramic-natural enamel combinations, nanofilled composite resin caused less wear on natural teeth compared to ceramic antagonists. Additionally, Abbasi et al. [9], highlighted that wear behavior is influenced not only by chemical composition but also by manufacturing technique, brand, and material structure. This supports our observation of similar wear behavior between Isosit and NHC materials, suggesting that factors beyond mere composition, such as production quality and structural characteristics, play a significant role in wear resistance.

In this study, consistent with previous research [23,41], Isosit teeth exhibited significantly higher average wear values against porcelain antagonists. The similar wear values of NHC teeth against porcelain antagonists, comparable to Isosit teeth, may be attributed to the structural similarities between them. Although NHC teeth were developed to offer physical advantages, such as color and polish stability [7], they did not demonstrate superior wear resistance compared to Isosit teeth, despite their higher cost.

In vivo studies on the wear resistance of resin-based artificial teeth have also yielded inconsistent findings. Research has been unable to identify significant differences in the wear behavior of various artificial tooth materials [37,41]. In contrast, Ohlmann et al. (2007) reported that antagonist materials significantly influence the wear resistance of artificial teeth [35]. Therefore, it is evident that comprehensive analysis is necessary to provide reliable in vivo data and to correlate the clinical wear performance of resin-based artificial teeth with in vitro findings.

The limitations of this study include the fact that it was conducted under controlled in vitro conditions, which may not fully replicate the complex and dynamic environment of the oral cavity. Additionally, only a two-body wear test was performed, and three-body wear mechanisms were not evaluated; therefore, the study may not encompass all aspects of clinical wear behavior. The use of 10 specimens per group represents a limited sample size, which may restrict the generalizability of the results. Finally, distilled water at 35.6 °C was used during testing; however, the chemical and physical properties of natural saliva could not be replicated in the in vitro environment, representing another limitation.

## 5. Conclusions

Within the limitations of this in vitro study, the following clinical implications can be drawn:

1. Porcelain as an antagonist material caused the highest wear on both isosit and nanohybrid composite (NHC) artificial teeth, indicating caution when used in removable dentures, especially against softer materials.
2. Isosit and NHC artificial teeth showed similar wear resistance, suggesting both materials are viable choices depending on restorative needs.
3. Using the same material bilaterally (e.g., isosit–isosit or NHC–NHC) tended to reduce wear, which may help maintain occlusal stability over time, although this was not statistically significant.
4. Dental practitioners should aim to select compatible opposing materials, as matched materials across arches may reduce occlusal wear and extend prosthesis longevity.
5. Future clinical studies are necessary to evaluate the long-term wear behavior of these materials in functional oral environments, including scenarios with porcelain antagonists in both arches.

## Figures and Tables

**Table 1:** Descriptive statistics of wear values (mm^3^).

Antagonist Materials	Testing Materials	Total Volume Difference (mm^3^)					
		*n*	Mean	Standard Error of Mean	Standard Deviation	Minimum	Maximum
Isosit	Isosit	10	199.08	29.82	94.29	105.44	368.50
	NHC	9	222.40	31.59	94.78	113.30	356.71
	Total	19	210.13 ^A^	21.25	92.63	105.44	368.50
NHC	İsosit	10	217.95	26.06	82.40	109.42	355.72
	NHC	10	197.09	33.35	105.48	105.83	443.66
	Total	20	207.52 ^A^	20.73	92.74	105.83	443.66
Porcelain	İsosit	10	462.85	32.99	104.29	297.71	623.18
	NHC	9	479.34	37.78	113.35	306.57	621.35
	Total	19	470.66 ^B^	24.30	105.93	297.71	623.18

Different letters show differences between the mean values.

**Table 2:** Two-way ANOVA results for the comparison of wear values.

Source of Variation	SS	DF	MS	F	*p*-Value
Testing Materials	578	1	578	0.0585	0.810
Antagonist Materials	875,108	2	437,554	44.2836	0.000
Testing Materials * Antagonist Materials	5547	2	2774	0.2807	0.756
Error	513,798	52	9881		

* The interaction effect between testing materials and antagonist materials.

## Data Availability

The datasets used and/or analyzed during the current study are available from the corresponding author on reasonable request.
